# Women Living with HIV in Zimbabwe: Their Stigma-Related Emotional Life and Sense of Self

**DOI:** 10.3390/ijerph22030364

**Published:** 2025-03-01

**Authors:** Limkile Mpofu, Elias Mpofu, Azwihangwisi H. Mavhandu-Mudzusi

**Affiliations:** 1 Department of Psychology, College of Human Sciences, University of South Africa (UNISA), Pretoria 0002, South Africa; 2 The Research Division, Jaylee Group Research Company, Pretoria 0002, South Africa; 3 Department of Rehabilitation and Health Services, University of North Texas, Denton, TX 76203, USA; 4 Department of Behavioural Health and Social Sciences Research, School of Health Sciences, University of Sydney, Sydney 2060, Australia; 5 Department of Educational Psychology, Faculty of Education, University of Johannesburg, Johannesburg 2006, South Africa; 6 Department of Social Sciences, College of Human Sciences, University of South Africa, Pretoria 0002, South Africa; mmudza@unisa.ac.za

**Keywords:** HIV-positive rural women, interpretive phenomenological analysis, stigma and discrimination, emotional life, sense of self

## Abstract

This study explored women living with HIV (WLHIV)’s stigma-related emotional life and sense of self in a rural Zimbabwean setting. The objective of this study was to understand the sense of stigma in the emotional lives and self-perception of women living with HIV in rural Zimbabwe. The participants were a purposive sample of 20 rural women living with HIV. Their age ranged from 20 to 65 years old. WLHIV completed semi-structured individual interviews on their emotions and sense of life. The interpretive phenomenological analysis (IPA) revealed that these rural women living with HIV endure humiliation and isolation, leading them to feeling hopeless. Their society (significant others) perceived them as burdensome social others from which little could be expected. These women experience this sense of “otherness” that represents them as social outcasts, which results in a deep sense of social isolation and loneliness, worthlessness, withdrawal, and hopelessness. The women self-perceived themselves to be constantly managing their sense of dehumanization and being stereotyped as primarily with an identity defined by disease or illness by society. The findings suggest a need for the development and implementation of support programs for building healthy self-identities for women living with HIV. Such programs would focus on strategies that counteract societal and self-stigmatization living with HIV and AIDS for full community inclusion.

## 1. Introduction

Women living with HIV may be stigmatized as failures in social roles, from being characterized as essentially immoral [1]. Goffman defined stigma is “an attribute that is deeply discrediting, that reduces the bearer from a whole person to a tainted, discounted one” [2] (p. 3). It risks adverse psychosocial and emotional consequences [3]. Previous studies documented demeaning and discriminatory practices against people living with HIV [4,5]. Yet, few studies explored these anti-HIV/AIDS social attitudes among women living with HIV in collectivistic sub-Saharan African cultures, where interdependence is highly valued and beyond a person’s disease status. Studies on gendered HIV stigma in collectivist societies would provide evidence for culturally sensitive interventions for supporting the full community inclusion of women living with HIV (WLHIV), particularly in rural communities where people have stronger family ties. This study explored the lived stigma experiences of Zimbabwean women living with HIV.

### 1.1. The Zimbabwean Gendered HIV Context

Zimbabwe has had a considerable reduction in the prevalence of HIV/AIDS among its population [6,7,8]. However, gendered HIV-related stigma remains an issue for WLHIV, and the risk is higher for those in rural communities. In this context, everyone is related to everyone else by blood, and health information is shared among kinsfolk. Regardless, women are at a higher risk for HIV-related stigma because their family roles are a lot more public compared to males, and women also consult with local clinics more than their male counterparts for their various sexual and reproductive health needs. From these interactions, which may include HIV testing if they were expecting, word about their HIV status may be leaked by clinic staff in their social circles [9]. In the Zimbabwean context, women are often assumed to have contracted HIV through promiscuity or prostitution. However, in actuality, they would have been infected through heterosexual and monogamous relationships [10]. Such attributions risk replacing these women’s identities with an overly disease-focused social identity in their community. At the same time, women might want to be aligned and treated as women and mothers [10]. This would be despite the fact that the women would have established cultural identities as good mothers who prioritize the protection of their children regardless of the social threats of HIV-related stigma [1,11].

According to Mercurio and Landry [12], shame is a self-conscious, negative emotion that involves experiencing the desire to hide, disappear, or die. Such a sense of personhood occurs when individuals evaluate themselves in relation to some internalized standard and determine that they have failed to meet that standard. In this regard, women’s rights continue to be violated through cultural practices that portray women as minorities while demeaning the women and denying them their subject positions as decision-makers. Women’s empowerment is critical to curbing HIV-related stigma.

### 1.2. Goal of the Study

The aim of this study is to explore the HIV-related stigma, emotional life, and sense of self of Zimbabwean women living with HIV. It addressed two questions as follows:

1. What emotional qualities characterize women living with HIV/AIDS?
2. How do women living with HIV self-perceive their sense of personhood through their self-esteem and self-efficacy?

The findings could assist the development and implementation of support programs for building healthy self-identities for women living with HIV needs.

## 2. Materials and Methods

### 2.1. Research Design

This study utilized an interpretive phenomenological inquiry [13] to understand WLHIV’s emotional experiences and senses of self. The interpretive phenomenological inquiry refers to the “unearthing of the phenomena from how people interpret and attribute meaning to their existence” [14] (p. 1). It was adopted for this study because of its sense-making approach to people’s life experiences. Interpretive phenomenological inquiry allows for a nuanced explication of participants’ experiences.

### 2.2. Participants and Setting

The participants were a purposive sample of women living with HIV (*n* = 20) from rural Matabeleland South Province of Zimbabwe. Their ages ranged from 20 to 65 years old. Regarding their relationship status, 5 were single, 7 were married, 2 were divorced, and 6 were widowed. They were all on antiretroviral (ARV) therapy and were permanent residents of their rural villages. The sample size was determined based on the principle of data saturation, common in qualitative IPA studies exploring deeply personal experiences. According to Smith, IPA is committed to examining experience in its complexity and uncovering what happens when a lived experience takes on a particular significance for people [15].

### 2.3. Data Collection

The participants completed semi-structured face-to-face interviews on their stigma-related emotional life and sense of self as WLHIV. The interview questions focused on the women’s emotions and sense of self, on how the women living with HIV self-perceived their sense of personhood through their self-esteem and self-efficacy. Sample questions were as follows: “Tell me about your experiences as a woman (personhood) living with HIV in this village. Tell me the difficulties or bad situations you encountered in this village, your emotions, and your sense of self”. For the trustworthiness and credibility of the data, triangulation techniques were employed, such as cross-checking the coherence of field notes and member checks with a subsample of the interviewees.

### 2.4. Procedure

The research ethics committee of the University of South Africa and the Medical Research Council of Zimbabwe approved this study. The participants consented to this study. They were informed and assured of the confidentiality of the data they provided and their right to discontinue the interviews at any time without penalty. Interview data were de-identified for the analysis. Participants could answer the interview questions in their preferred language (Ndebele or English).

### 2.5. Data Analysis

The data were systematically analyzed by following the procedures by [13,16], comprising the steps of (1) immersing oneself in the original data through reading and rereading the narratives, (2) observing and commenting by scripting any emerging themes, (3) applying in-depth inductive qualitative analysis, and (4) generating initial codes. Final themes were constructed utilizing the interpretive phenomenological analysis [13]. For the dependability of interpretations, a collaborator created a summary list of themes from margin notes, developing the emergent themes using the transcripts. Differences were resolved by discussion for consensus.

## 3. Findings and Discussion

Thematic analysis identified three themes: (i) dehumanizing behavior, (ii) hopelessness/worthlessness, and (iii) social isolation/withdrawal/loneliness. Table 1 presents the summary themes and representative quotes (Table 1). In the sections to follow, each theme is discussed with excerpts from the female participants.

### 3.1. Theme 1: Dehumanizing Behavior

WLHIV reported a sense of being dehumanized by significant others, such as blamed and shamed for their HIV infection. What annoyed the women more was that the perpetrators of such behavior were their loved ones, which included partners, close family members, in-laws, friends, and the community.

For instance, a 42-year-old married woman said she got hurt and very angry when recalling the blame and name-calling by her mother-in-law, who, in the previous years before she knew her status, was very close to her. The mother-in-law now saw her as unfit to raise her children.

*My mother-in-law called me defiant and uncaring and said that I had brought HIV to our matrimonial home. She even told my children that I killed my husband and that I was insensitive toward my family*.

(Respondent # 04, 42 years, married)

Another 39-year-old married woman expressed anger and said she was stressed by her society, which had given her a label and called her by her HIV status.

*This community is a nuisance. How can they label me like that? Am I the first person to be HIV positive? When I go out, other women point fingers at me and call me a prostitute.*

(Respondent # 20, 39 years, married)

There were also unmarried women living with HIV in this community. These women also expressed annoyance at how they were devalued and subjected to several harmful negative stereotypes by their community, making the women even doubt themselves. A 38-year-old unmarried woman shared her experience as follows:

*This community gossips behind me. They make me doubt myself. They say many things, that I am a prostitute and that I won’t get a suitor because of my HIV-positive status. I wonder if I will ever get someone to marry me*.

(Respondent # 09, 38 years, single)

For sero-discordant couples, the dehumanizing behavior came in the form of social rejection, where the HIV-negative man would divorce their wives for being HIV-positive.

The following quote from an irritated 38-year-old divorced woman bears evidence:

*I will never forgive my husband; he rejected me just because I tested positive. I have no idea why since I know no other man besides him. He divorced me and claimed that he could not continue his life with an HIV-positive woman. I am so irritated by this man. He seems to have forgotten everything I have done for him as a loyal wife. This is so troubling, eish!*

(Respondent # 19, 38 years, divorced)

From the narration above, women experienced psychological distress managing relations and their lifelong illness. In this study, unmarried women living with HIV, in particular, were reported to be devalued and subjected to several harmful negative stereotypes by their community. These findings are similar to those of related studies from sub-Saharan Africa, such as those by [17]. In this study, the cumulative effects of HIV-related stigma and pre-existing prejudices in relation to gender, marital status, rejection, and labeling collectively increased women’s psychological burden. According to Mpofu and Ganga-Limando [18], these rural women are at risk of developing (or being labeled as having) chronic psychopathology or maladaptive behaviors, as they require unique mental and physical health needs reflective of their experiences and journeys with HIV.

### 3.2. Theme 2: Hopelessness/Worthlessness

The women reported a sense of hopelessness due to the social prejudice and discrimination against them, from which experiences they developed internalized stigma associated with lower self-esteem, self-efficacy, and psychological stress. The following statements from a 36-year-old, 44-year-old, 50-year-old, and 49-year-old women bear evidence:

*My future was bleak after testing HIV positive. I am not going to make it and see my grandchildren. I am so worried I might die any time. I take these antiretroviral drugs, but for how long?*

(Respondent # 02, married, 36 years)

A 44-year-old married woman living with HIV, who was sent back to her village by her in-laws after the death of her husband, recounted the following:

*I am so depressed and feel useless right now, even at 44 years old. I am not that old, you know, but look at me. I am back at my home family after my matrimonial family sent me back here after the death of my husband. Eish, I am doomed.*

(Respondent # 13, 44 years, married)

Some women felt worthless, with some feeling a sense of being good for nothing or useless or valueless due to their HIV-positive status or the social prejudice and discrimination against women living with HIV. Such feelings were associated with suicidal ideation, as narrated by a 44-year-old woman.

*Why am I still breathing> I want to die and escape the misery of this world. I have been sick for some time. It’s just on and off. My husband is better off because he died long back and is resting now. What about me?*

(Respondent # 17, 44 years, widowed)

A 50-year-old married woman had to say the following:

*This HIV-positive diagnosis impacted my life. I feel sad and want to die, at least.*

(Respondent # 11, 50 years, married)

Another 49-year-old widowed woman shared her experience of feeling hopeless and worthless due to her HIV status as follows:

*What’s the importance of living when you have HIV? I am worthless. Now, my life is bordering on this disease. It is too sad*.

(Respondent # 12, 49 years, widowed)

In related studies by Rich et al. [19] in Zimbabwe, the young adults’ experiences of self-stigma reportedly led to poor well-being and decreased mental and physical health. In their study, Rich and colleagues found that HIV-related negative self-perceptions were associated with self-stigmatizing beliefs that adversely affected these young adults’ quality of life [19]. With HIV-related stigma, the women would feel devalued within their family lives.

### 3.3. Theme 3: Social Isolation/Withdrawal/Loneliness

The women living with HIV in this study reported self-objectifying experiences, including a sense of loneliness and social isolation due to their HIV status or the social prejudice and discrimination against women living with HIV.

A 41-year-old married woman shared her experience with sadness:

*My self-esteem is crushed. I don’t interact with people in my community because of their negative behavior toward us people living with HIV. It is painful, indeed. I will eventually leave this community to escape the shame and judgment they display on us. It’s torture to be reminded of your status every day. I am happy alone behind closed doors*.

(Respondent # 11, 41 years, married)

A 32-year-old unmarried woman distasted mingling with society because of her appearance anxiety (the state of knowing that she is HIV positive).

*Their eyes could kill me. They thought the worst of me. It sounds as though I could hear the word promiscuity, and this community would look at you as if you are disgusting. It’s okay; I just had to withdraw from such a community.*

(Respondent # 08, 32 years, single)

Some of the WLHIV reported to have stopped their small business enterprises from the emotional overloading living with HIV-related stigma. For instance, a 38-year-old single woman said the following:

*I stopped my chicken business because of people’s behavior toward me after testing HIV positive. You know, I was breeding and selling chickens in my village. I was doing well in my business, but things changed after people became aware of my HIV status. I could not continue because of their name-calling, and others avoided me. So, I completely withdrew from people and stopped my business.*

(Respondent # 16, 38 years, single)

The women displayed low self-esteem as an indicator of their affected well-being as they judged their overall self-worth, contributing to their maladaptive social behaviors. For example, the participants expressed their pain from isolation and loneliness as follows:

*It’s quite painful to live like this in this village. I feel lost; no one visits me, and I have no one to talk to. My homestead is lonely; my people have deserted me, and I have no one to turn to during difficult times. All my people who used to care are gone, my children, my husband, they all died. My relatives feel I am a burden, and they don’t come to visit me.*

(Respondent # 7, 59 years, widowed)

In this study, we see some women running out of options and self-imposed social isolation on themselves in a bid to escape HIV-related stigma. This may be an internalized HIV-related stigma effect regarding the way they struggle to access social support because they fear social rejection [20,21]. In a South African study, Wouters et al. [22] reported that social relationship impairment hampers successful psychological adaptation to HIV (see also [23]).

## 4. Implications for Practice

In African communities, society assigns roles and responsibilities to women’s positions in the family and community, while biology also confirms the differences between men and women [24]. This implies that the women would have to assess their physical, psychological, and social risks in order to guard against any stigma-related emotions to be self-caring. Based on these findings, there is a need for interventions to optimize and promote women’s health by encouraging community involvement and participation in WLHIV. This will require strengthening the implementation of gender-friendly policies and programs at the village level that increase their access to HIV education and information. Peer support networks or gender-sensitive counseling tailored to destigmatize WLHIV would be important, particularly for rural women in patriarchal societies like Zimbabwe. This would entail capacity-building initiatives on stigma reduction that utilize empathetic communication for healthcare providers to address stigma.

### Limitations and Future Directions

One of the limitations of this study is that it is based on a rural community in Matabeleland South Province, Zimbabwe, and the findings may be different in other locations. Studies should seek to replicate these findings in other locations from within the same or a different rural community. Moreover, a mixed methods approach would help to triangulate the findings from this study.

## 5. Conclusions

In the present study, rural WLHIV in Zimbabwe reported experiencing stigma and a sense of dehumanization. From these experiences, they reported emotional distress affecting their mental health.

The women’s narration and discussion suggest a need for gender-sensitive health promotion policies and programs to mitigate the emotional toll of HIV-related stigma on rural women This is a clear call to action for policymakers, researchers, and community leaders to develop gender-sensitive group interventions to decrease women’s isolation, sense of worthlessness, and individualization of health problems among WLHIV and, instead, encourage spaces for collective support, action, and change to guide future stigma-reduction efforts.

## Figures and Tables

**Table 1:** Summary of themes with quotes and key findings.

Themes	Representative Quotes	Key Findings
Theme 1: Dehumanizing behavior	*My mother-in-law called me defiant and uncaring and said that I had brought HIV to our matrimonial home. She even told my children that I killed my husband and that I was insensitive toward my family* (Respondent # 04, 42 years).	The women were blamed and shamed for being HIV-positive, rejected, and subjected to undignified treatment and gender stereotypes. The women were devalued and subjected to several harmful negative stereotypes by their community. The women “self-stigmatized” themselves as they cognitively and emotionally absorbed the stigmatizing assumptions and stereotypes about their HIV status, and they came to believe and apply the stigma to themselves. The women were negatively affected psychologically.
	*This community is a nuisance. How can they label me like that? Am I the first person to be HIV positive? When I go out, other women point fingers at me and call me a prostitute. (Respondent # 20, 39 years)*	
	*This community gossips behind me. They make me doubt myself. They say many things, that I am a prostitute and that I won’t get a suitor because of my HIV-positive status. I wonder if I will ever get someone to marry me* (Respondent # 09, 38 years)	
	*I will never forgive my husband; he rejected me just because I tested positive. I have no idea why since I know no other man besides him. He divorced me and claimed that he could not continue his life with an HIV-positive woman. I am so irritated by this man. He seems to have forgotten everything I have done for him as a loyal wife. This is so troubling, eish!* (Respondent # 19, 38 years).	
Theme 2: Hopelessness /Worthlessness	*My future was bleak after testing HIV positive. I am not going to make it and see my grandchildren. I am so worried I might die any time. I take these antiretroviral drugs, but for how long?* (Respondent # 02, 36 years).	The women in this study had no hope for their future. Their internalized stigma was associated with a number of negative outcomes, including lower self-esteem, self-efficacy, and recovery orientation, as well as greater psychological stress. Women felt worthless, with some feeling a sense of being good for nothing or useless or valueless due to their HIV-positive status or the social prejudice and discrimination against women living with HIV. Such feelings were associated with suicidal ideation. Adverse psychological disorders such as depression, anxiety, stress, anger, sadness, loneliness, hopelessness, and reduced physical well-being impacted the mental health of women living with HIV.
	*I am so depressed and feel useless right now, even at 44 years old. I am not that old, you know, but look at me. I am back at my home family after my matrimonial family sent me back here after the death of my husband. Eish, I am doomed.* (Respondent # 13, 44 years).	
	*Why am I still breathing> I want to die and escape the misery of this world. I have been sick for some time. It’s just on and off. My husband is better off because he died long back and is resting now. What about me?* (Respondent # 17, 44 years)	
	*This HIV-positive diagnosis impacted my life. I feel sad and want to die, at least.* (Respondent # 11, 50 years)	
	*What’s the importance of living when you have HIV? I am worthless. Now, my life is bordering on this disease. It is too sad* (Respondent # 12, 49 years).	
Theme 3: Social isolation /Withdrawal /loneliness	*My self-esteem is crushed. I don’t interact with people in my community because of their negative behavior toward us people living with HIV. It is painful, indeed. I will eventually leave this community to escape the shame and judgment they display on us. It’s torture to be reminded of your status every day. I am happy alone behind closed doors* (Respondent # 11, 41 years).	Women displayed some shameful feelings about being HIV-positive, and this led them to withdraw from society and be isolated. Thus, internalized stigma was detrimental to these women as it led to emotional withdrawal. Self-removal from interaction with significant others in the community due to their HIV status or the social prejudice and discrimination against women living with HIV. Women living with HIV found it difficult to interact with significant others because of the social prejudice against them by members of their communities. They turned to social isolation or withdrawal to avoid social discrimination by significant others in their communities. The rural women living with HIV evaluated themselves in relation to some internalized standard and determined that they failed to meet that standard they had set for themselves.
	*Their eyes could kill me. They thought the worst of me. It sounds as though I could hear the word promiscuity, and this community would look at you as if you are disgusting. It’s okay; I just had to withdraw from such a community.* (Respondent # 08, 32 years)	
	*I stopped my chicken business because of people’s behavior toward me after testing HIV positive. You know, I was breeding and selling chickens in my village. I was doing well in my business, but things changed after people became aware of my HIV status. I could not continue because of their name-calling, and others avoided me. So, I completely withdrew from people and stopped my business.* (Respondent # 16, 38 years)	
	*It’s quite painful to live like this in this village. I feel lost; no one visits me, and I have no one to talk to. My homestead is lonely; my people have deserted me, and I have no one to turn to during difficult times. All my people who used to care are gone, my children, my husband, they all died. My relatives feel I am a burden, and they don’t come to visit me.* (Respondent # 7, 59 years)	

## Data Availability

The data that support the findings of this study are available upon request from the corresponding author, L.M.
